# Predicting Time on Prolonged Benefits for Injured Workers with Acute Back Pain

**DOI:** 10.1007/s10926-014-9534-5

**Published:** 2014-08-28

**Authors:** Ivan A. Steenstra, Jason W. Busse, David Tolusso, Arold Davilmar, Hyunmi Lee, Andrea D. Furlan, Ben Amick, Sheilah Hogg-Johnson

**Affiliations:** 1 Institute for Work & Health, 481, University Ave, Suite 800, Toronto, ON M5G 2E9 Canada; 2 Institute for Work & Health, Toronto, ON Canada; 3 Dalla Lana School of Public Health, University of Toronto, Toronto, ON Canada; 4 Departments of Anesthesia and Clinical Epidemiology & Biostatistics, McMaster University, Hamilton, ON Canada; 5 Department of Medicine, Faculty of Medicine, University of Toronto, Toronto, ON Canada; 6 Toronto Rehabilitation Institute, University Health Network, Toronto, ON Canada; 7 Department of Health Policy and Management, Robert Stempel College of Public Health & Social Work, Florida International University, Miami, FL USA

**Keywords:** Back pain, Prognosis, Workers’ compensation, Occupational health, Insurance, Disability

## Abstract

*Introduction* Some workers with work-related compensated back pain (BP) experience a troubling course of disability. Factors associated with delayed recovery among workers with work-related compensated BP were explored. *Methods* This is a cohort study of workers with compensated BP in 2005 in Ontario, Canada. Follow up was 2 years. Data was collected from employers, employees and health-care providers by the Workplace Safety and Insurance Board (WSIB). Exclusion criteria were: (1) no-lost-time claims, (2) >30 days between injury and claim filing, (3) <4 weeks benefits duration, and (4) age >65 years. Using proportional hazard models, we examined the prognostic value of information collected in the first 4 weeks after injury. Outcome measures were time on benefits during the first episode and time until recurrence after the first episode. *Results* Of 6,657 workers, 1,442 were still on full benefits after 4 weeks. Our final model containing age, physical demands, opioid prescription, union membership, availability of a return-to-work program, employer doubt about work-relatedness of injury, worker’s recovery expectations, participation in a rehabilitation program and communication of functional ability was able to identify prolonged claims to a fair degree [area under the curve (AUC) = .79, 95 % confidence interval (CI) .74–.84]. A model containing age, sex, physical demands, opioid prescription and communication of functional ability was less successful at predicting time until recurrence (AUC = .61, 95 % CI .57, .65). *Conclusions* Factors contained in information currently collected by the WSIB during the first 4 weeks on benefits can predict prolonged claims, but not recurrent claims.

**Electronic supplementary material:**

The online version of this article (doi:10.1007/s10926-014-9534-5) contains supplementary material, which is available to authorized users.

## Introduction

Work disability due to back pain (BP) is a multidimensional problem [1] associated with high compensation and treatment costs. Total costs of BP in Canada are estimated to be between $11 billion and $23 billion per year [2]. In a US study, workers with BP recurrences accounted for 71.6 % of the total costs of BP [3, 4]. Costs associated with productivity losses due to BP (indirect costs) are estimated to be 85 % of total costs in the general population [5] and even higher in work related BP [6]. Workers who are at low risk for chronic disability will most likely return to work (RTW) with limited assistance [7]. Those at high risk for chronic disability may benefit from tailored interventions [8]. If so, the burden of BP could be reduced through the early identification of those at risk of chronic disability among those away from work due to BP.

A number of studies have been published on prognosis in RTW following BP, either with an explanatory [9] or predictive focus [10]. Our research objective was to develop a prediction tool. A prediction tool is based on the most parsimonious model that accurately predicts outcomes in a generalizable manner.

Work-related BP is a multidimensional problem; therefore, predictive factors should be collected from several key actors [workplace partners, health-care providers (HCPs), injured workers, insurers] to capture the complex interactions that influence outcomes [1]. Yet most of the existing literature relies on information gathered from injured workers, which is often limited to clinical factors. Information from several key actors should be considered to achieve a prognostic model that explains more variability in outcomes than previous studies [11].

Researchers who develop prediction tools should consider who the key users of their tool will be and take their perspectives into account. For example, 4 weeks post-injury is a key decision-making point for case management within Ontario’s Workplace Safety and Insurance Board (WSIB). At this time point, case managers want to know how long it will likely take for an injured worker to RTW, and whether the likelihood of a recurrence is high or low [12]. As such, information available within the first 4 weeks of a claim should be used in building a predictive model for workers’ compensation insurers such as the WSIB [13].

Our study objective was to build prediction rules for time on disability benefits and time until recurrence for workers with lost-time claims (LTCs) secondary to BP. Two research questions were considered:

1. What combination of factors measured within the first 4 weeks of a BP-related WSIB claim best predicts the length of the first episode of wage-replacement benefits?
2. After a first episode of being on BP-related benefits has ended, what combination of factors captured within the first 4 weeks of the first episode best predicts the length of time until a recurrence?

## Methods

The development of a prediction tool includes three steps [14]: (1) identifying factors with predictive power (derivation); (2) establishing the strength and accuracy of the factors in different settings (validation); and (3) examining if the tool improves outcomes and/or reduces costs (impact analysis). Answering the two research questions above addresses the first two steps in this three-step process: derivation and internal validation.

### Study Sample

A random sample of 6,657 injured workers was taken from among all (n = 18,974) injured workers in Ontario, Canada, who reported an uncomplicated back injury (strain or sprain) with a date of injury between January 1 and June 30, 2005. Follow up was 2 years after first day of injury [15]. Our prediction model was focused on information collected during the first 4 weeks of a claim. As such, we excluded workers if their claim was initially registered as a no-lost-time claim but later transitioned to a LTC claim, if their claim ended before 4 weeks, or if the number of days between the injury date and registration date of their claim was >30 days. The Health Sciences Research Ethics Board of the University of Toronto approved our study protocol.

### Sources of Data

To increase the feasibility of implementing prognostic models in practice, we built our models using data routinely collected in Ontario by the WSIB. Three sources of data were available through the WSIB for this purpose: (1) data from the WSIB electronic claim file database; (2) data from the electronic health-care billings database; and (3) data collected through forms filled out by employers, workers and HCPs available as imaged files in an electronic database.

When a claim for wage-replacement benefits is submitted to the WSIB, the employer, worker and HCP are asked to complete a number of forms. The employer form (Form 7) is mandatory and must be submitted within 3 days of a work-related injury. Late or incomplete reporting can lead to a fine. The worker may elect to fill out a Form 6 on a voluntary basis if he or she has expenses related to the workplace injury and/or expects the employer has not sent in Form 7. The HCP can elect to complete a Form 8 on a voluntary basis to support the patients’ claim that his or her injury is work-related (a prerequisite for receiving wage-replacement benefits through the WSIB). The WSIB requests HCPs to complete and submit a Functional Abilities Form (FAF) for each claim, and provides reimbursement as incentive. During the data-collection phase of this study, two different versions of Form 8 were in use: a version introduced in 1999 that was still in use by some HCPs in 2005, and a newer version introduced in 2003. These forms differed slightly in the factors collected. The WSIB decides on work relatedness of the injury based on the available information from these sources. When crucial information is missing this decision can be delayed.

An experienced analyst extracted and assembled data elements from the WSIB’s claim file database and health-care billings database. Data extractors accessed Forms 6, 7 and 8 through WSIB’s imaged files and saved the information into an Access database. In order to minimize data-entry mistakes, data entry forms were built that included range checks and missing-value alerts. For the first 100 cases, all data was entered independently and in duplicate by two abstractors and then compared using the PROC COMPARE procedure in SAS 17. This comparison revealed 98 % agreement; therefore, only a single abstractor completed data entry for the remaining cases.

### Candidate Predictors

When building a prediction rule, selection of candidate predictors should be informed by evidence from the literature and consultations with content experts [14, 16]. Guided by this strategy, we chose our independent variables based on a systematic review that explored factors associated with RTW in workers in the acute phase of BP-related work disability [17] and on stakeholder input. We then predicted the direction of anticipated effects (Table 1).

**Table 1:** Overview of all variables selected and the source of information

Construct	Source	Level of evidence	Direction of effect
Worker-related factors			
Age	Electronic claim file	Inconsistent findings in multiple studies (due to non report)	Less likely to RTW if older
Sex	Electronic claim file	Insufficient evidence	No effect
Presence of language barriers	Electronic claim file	Insufficient evidence (not enough studies)	Less likely to RTW
Prior work absence	Electronic claim file and imaged files	Moderate evidence	Less likely to RTW
Recovery expectations of worker	Form 8	Strong evidence	Less likely to RTW if low
Physical functioning, functional ability	Form 8	Strong evidence	Less likely to RTW if more disabled
Work-related factors			
Physical demands	Electronic claim file	Strong evidence	Less likely to RTW when high
Job tenure	Electronic claim file	Moderate evidence	Less likely to RTW if shorter
Modified duties	Form 7	Strong evidence	More likely if modified
Union member	Form 6	Insufficient evidence (not enough studies)	More likely to RTW if member
Health-care-related factors			
Treating health-care provider	Health-care billings	Strong evidence	More likely to RTW when treated by some HCPs
WSIB work rehabilitation program	Health-care billings	Stakeholder input	More likely to RTW
Early and prolonged prescription of opioids	Health-care billings	Insufficient evidence (not enough studies)	Less likely to RTW
Insurer-related factors			
Benefits paid: height of compensation, employer continues pay	Form 7	Moderate evidence	Less likely to RTW if higher compensation
Process of RTW			
Worker signature	Form 7	Moderate evidence	More likely to RTW
HCP discussed RTW	Form 8		
Communication of functional ability to RTW	Health-care billings		
Doubt of work-relatedness	Form 7	Insufficient evidence (not enough studies)	Less likely to RTW

This table is based on a systematic review of similar studies [17]

#### Worker-Related Factors

We established whether workers faced a language barrier [18] based on requests by the employer or worker for services in languages other than English or French. Prior work absence [17] was based on previous lost-time and health-care only (i.e. no-lost-time) claims in the WSIB claim database. Both types of claims were examined, separately and together. Worker report (Form 6) of a previous similar injury or previous claim (either in Ontario or in another jurisdiction) was also examined as a source of information.

The 1999 version of Form 8 required HCPs to record their recovery expectations with respect to their patients (claimants). We coded this factor as missing when HCPs completed the 2003 version of Form 8, as the item asking about recovery expectations was not present in the newer version of the form.

We acquired information on claimants’ functional abilities [13, 17] through their HCPs’ yes/no responses to three questions: one regarding patient limitations related to RTW, another on the ability of a patient to operate a motor vehicle and another on the ability of the patient to use public transport.

#### Work-Related Factors

Workplace physical demands [17] were classified based on the National Occupational Code as manual (high physical demands), mixed or non-manual work [19]. Job tenure was classified as the number of years of experience in the job [18, 20]. Both workers’ reports (Form 6) and employers’ reports (From 7) provided information on union membership, but we considered workers’ reports of union membership as the most reliable source of information.

#### Health-Care-Related Factors

Injured workers could have health-care provider expenses covered for using any one of three types of provider: medical doctor, physiotherapist or chiropractor. We coded WSIB-compensated expenses for each of these HCPs and entered each factor in our prediction models. When we created a variable for WSIB-compensated prescriptions for opioids in the first 4 weeks of a claim, we combined weak and strong opioids.

#### Insurer-Related Factors

We used Form 6 to determine level of benefits paid. Benefits paid by the WSIB in Ontario are 85 % of net earnings prior to injury, but some employers report (as requested in Form 6) that they continue to pay salary regardless of the injury, which results in a higher income for some workers.

#### The Return-to-Work Process

The process preceding timely and safe RTW is considered important to its sustainability [21, 22]. Therefore, a number of RTW process elements were examined. Worker involvement in the RTW process was assessed by: (1) registering the worker signature on Form 6 (workplace form); (2) examining the HCP’s report (on Form 8) indicating whether or not RTW was discussed with the worker; and (3) examining the number (from none to more than four) of FAF sent by the HCP as requested by the WSIB to communicate the worker’s functional ability to RTW. The employer’s report (Form 7) indicating doubt about the work-relatedness of an employee’s back injury was used as a proxy for a possible adversarial process [23].

### Outcome Measures

We used two outcomes to characterize the claim disability process over the first 2 years [24]:

1. time on benefits during the first BP episode, which was the length in calendar days of the first continuous episode of full wage replacement; and
2. time until recurrence after the first BP episode: which was the length in calendar days from the end of the first episode of wage replacement to the start of the second episode of full wage-replacement benefits for the same injury.

### Statistical Analyses

#### Derivation of the Model

We performed bivariate analyses for all potential prognostic factors with each outcome measure. All independent variables that demonstrated an association with an outcome (at *P* < .20) were entered into our multivariable models. Both models were adjusted for age and sex, and our model exploring factors associated with recurrence was also adjusted for time on benefits in the first episode as the number of days, a continuous variable. We estimated the mean and median number of days for each outcome using the Kaplan–Meier procedure in IBM SPSS version 19.

Although multiple imputation for missing data has become a standard practise in prediction tool development [25], we elected to designate ‘missing’ as a distinct category. First, for practical reasons, users of the predictive tool (e.g. WSIB case managers) will be faced with missing data in practice. Second, our data were poorly suited for multiple imputation due to low multiple correlation coefficients between available and missing variables.

All outcomes were lengths of time, with censoring at 2 years’ post injury. We used Cox semi-parametric modeling to examine the relationship between multiple predictors and outcomes and checked the proportional hazards assumption [26]. We used an automated backward selection procedure to build all multivariable models. For all predictive factors, the reference category is indicated with a hazard rate ratio (HRR) of one. An HRR smaller than one indicates longer time until end of benefits or a longer time until recurrence. Interaction terms between age and workplace accommodation and prior sick leave and workplace accommodation were added based on previous research [27] to determine whether the model could be improved [26].

#### Internal Validation

We used bootstrapping techniques to validate our prediction model; i.e. to adjust the estimated regression coefficients for over-fitting and the model performance for over-optimism [16]. Variables that had a bivariate association with the outcome of *P* < .20 were selected for internal validation. The bootstrapping results are presented as the percentage of 2,000 bootstrap samples in which a particular factor was present in the final multivariable model. Factors that were present in more than 50 % of all 2,000 bootstrap models were included in the final model. We adjusted all models for age and sex, and also for time on benefits during the first episode if time until recurrence was the outcome. For bootstrapping, we used the statistical program R version 2.15.3—(The R project for statistical computing, www.r-project.org) [28].

#### Model Fit

We calculated the receiver-operating characteristic curve to assess each model’s ability to discriminate between workers for the outcome of interest. Benefit status at 180 and 720 days post-injury and the risk score (xBeta) as calculated for each injured worker from our validated models [29, 30] were compared. The risk score for each injured worker from our recurrences model was compared with a recurrence at 1, 3 and 6 months after end of the initial episode. The following criteria were used to evaluate the area under the curve (AUC): .90–1.0 = excellent, .80–.90 = good, .70–.80 = fair, .60–.70 = poor, <.50–.60 = fail [29]. Analyses were performed using SPSS 19 and R [28].

## Results

**Fig. 1:**
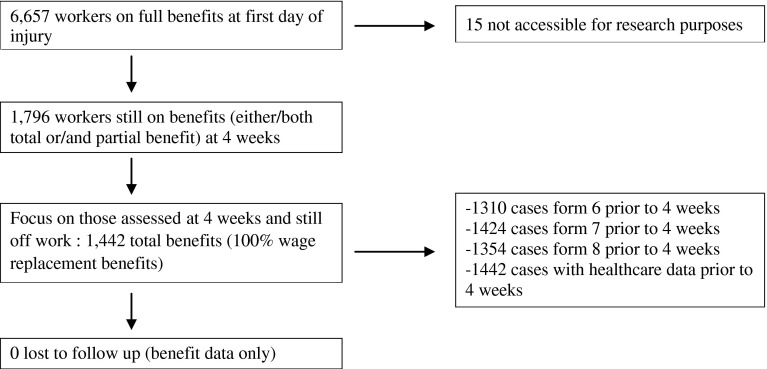
Flow chart of the study

From the sample of 6,657 cases, 15 cases were not accessible for research purposes and 1,442 were still on full benefits at 4 weeks and these provided data for our analyses (Fig. 1). All eligible workers had complete data for their duration on benefits and recurrence of benefits; however, 95 claims (6.6 %) remained on benefits continuously during the two-year follow-up and were not included in the analysis of recurrence. That left 1,347 cases at risk for a recurrence during follow-up. Baseline characteristics for all workers included in our analyses are reported in Tables 2 and 3. The mean age at time of work injury of the included workers (n = 1,442) was 41.3 years (SD 10.5).

**Table 2:** Variables associated with time on benefits (n = 1,442)

Variable	uHRR	*P* value	Proportion of bootstraps remaining in the model	aHRR
Age in categories				
15 to <25 (n = 97)	1.14 (.90, 1.44)	.001	1	1.27 (1.00, 1.60)
25 to <35 (n = 291)	1			1
35 to <45 (n = 486)	.89 (.77, 1.04)			.90 (.78, 1.05)
45 to <55 (n = 411)	.87 (.74, 1.01)			.84 (.72, 99)
55–65 (n = 157)	.64 (.52, .79)			.65 (.52, .80)
Men (n = 890)	1	.116	1	1
Women (n = 552)	1.09 (.98, 1.22)			.97 (.87, 1.09)
Previous lost-time claim		.114	.110	–
Yes (n = 657)	1.08 (.97, 1.21)			
No (n = 785)	1			
Previous no-lost-time claim	1.11	.066	.095	–
Yes (n = 750)	(.99, 1.23)			
No (n = 692)	1			
Physical demands		.007	.785	
Non-manual (n = 139)	1			1
Mixed manual (n = 465)	1.00 (.82, 1.21)			1.05 (.86, 1.28)
Manual (n = 798)	.83 (.69, .99)			.84 (.69, .1.01)
Missing (n = 40)	.94 (.65, 1.02)			.95 (.65, 1.02)
Language				
French/English (n = 1,396)	1	.06	.165	–
Other (n = 46)	.74 (.54, 1.01)			
Union member				
Yes (n = 610)	1.29 (1.15, 1.45)	.001	.790	1.14 (1.01, 1.29)
No (n = 656)	1			1
Missing (n = 176)	1.34 (1.13, 1.59)			1.34 (1.12, 1.60)
Early RTW program		.001	1	
Yes (n = 1,042)	1			1
No (n = 278)	.58 (.50, .67)			.59 (.516, .69)
Missing (n = 122)	.68 (.56, .83)			.70 (.56, .87)
Employer continued pay salary		.001	.305	–
No (n = 1,234)	1			
Yes (n = 181)	1.31 (1.11, 1.53)			
Doubt work relatedness	1	.062	.575	1
No (n = 1,051)	.94 (.80, 1.10)			.929 (.78, 1.08)
Yes (n = 195)	1.19 (1.01, 1.40)			1.18 (.99, 1.40)
Missing value (n = 180)	.71 (.42, 1.20)			.76 (.43, 1.35)
Missing form 7 (n = 16)	–			–
Worker signed				
Yes (n = 327)	.88 (.77, 1.00)	.080	.025	–
No (n = 1,085)	1			
Missing (n = 30)	.82 (.56, 1.19)			
Recovery expected				
Yes (n = 224)	1	.02	.795	1
No (n = 11)	.36 (.17, .76)			.43 (.20, .92)
Missing value (n = 38)	.68 (.47, .97)			.66 (.46, .94)
HCP report form ‘03^a^ (n = 1,169)	.83 (.72, .96)			.86 (.74, .99)
Use public transport		.167	.030	–
No (n = 13)	.65 (.35, 1.19)			
Yes (n = 209)	1			
Missing (n = 1,209)	.89 (.77, 1.04)			
Functional abilities form		.0001	.990	1
0 (n = 754)	1			
1 (n = 426)	1.24 (1.10, 1.41)			1.12 (.996, 1.27)
2 (n = 181)	1.40 (1.19, 1.66)			1.21 (1.02, 1.44)
3 (n = 57)	1.60 (1.22, 2.10)			1.43 (1.07, 1.89)
≥4 (n = 24)	2.44 (1.62, 3.67)			2.32 (1.53, 3.52)
Medical doctor				
No (n = 204)	1	.832	.185	–
Yes (n = 1,238)	1.02 (.87, 1.19)			
Chiropractor				
No (n = 1,209)	1	.117	.260	–
Yes (n = 233)	.89 (.77, 1.03)			
Physiotherapist				
No (n = 1,006)	1	.816	.220	–
Yes (n = 436)	1.01 (.90, 1.14)			
POC				
No (n = 1,171)	1	.102	.530	1
Yes (n = 271)	1.12 (.98, 1.29)			1.15 (1.00, 1.33)
Opioid prescription				
No (n = 1,306)	1	.001	.955	1
Yes (n = 136)	.63 (.52, .76)			.71 (.58, .86)

All assumptions were met (HR <1 means longer time until end benefits, reduced rate of ending benefits)

*uHRR* univariable hazard rate ratio, *aHRR* adjusted hazard rate ratio

^a^Form8v99 = the healthcare provider form version 1999. Form8v03 = the healthcare provider form version 2003

**Table 3:** Variables associated with time until recurrence (n = 1,347)

First block risk factors	uHRR	*P* value	Proportion of bootstraps remaining in the model	mHRR
First episode length beyond 4 weeks	1.00 (1.00, 1.00)		1.000	1.00 (1.00, 1.00)
Age in categories				
15 to <25 (n = 96)	.70 (.41, 1.19)	.572	1.000	.70 (.41, 1.19)
25 to <35 (n = 275)	1			1
35 to <45 (n = 461)	1.04 (.77, 1.40)			.97 (.72, 1.31)
45 to <55 (n = 382)	.99 (.73, 1.36)			.98 (.72, 1.34)
55 to <65 (n = 133)	.90 (.59, 1.38)			.86 (.57, 1.32)
Men (n = 821)	1	.031	1.000	1
Women (n = 526)	1.27 (1.02, 1.58)			1.36 (1.09, 1.70)
Previous claim				
Yes (n = 1,018)	1.24 (.96, 1.62)	.098	.355	
No (n = 329)	1			
Physical demands		.085	.725	
Non-manual (n = 132)	1			1
Mixed (n = 448)	1.11 (.73, 1.71)			1.12 (.73, 1.72)
Manual (n = 730)	1.45 (.97, 2.16)			1.55 (1.02, 2.34)
Missing (n = 37)	1.29 (.62, 2.75)			1.43 (.66, 3.08)
Opioid prescription				
No (1,231)	1	.030	.675	1
Yes (116)	1.47 (1.06, 2.06)			1.52 (1.09, 2.13)
Early RTW program				
Yes (n = 1,002)	1	.022	.395	
No (n = 237)	.65 (.47, .90)			
Missing (n = 94)	.83 (.53, 1.30)			
Functional ability forms				
0 (n = 687)	1	.021	.630	1
1 (n = 404)	1.33 (1.04, 1.70)			1.31 (1.02, 1.68)
2 (n = 176)	1.60 (1.18, 2.19)			1.58 (1.15, 2.15)
3 (n = 56)	1.35 (.79, 2.30)			1.26 (.74, 2.15)
4+ (n = 24)	1.71 (.84, 3.49)			1.45 (.71, 2.99)
Medical doctor				
No (n = 189)	1	.084	.080	
Yes (n = 1,158)	1.33 (.95, 1.87)			
Physiotherapist				
No (n = 806)	1	.299	.310	
Yes (n = 541)	1.12 (.90, 1.39)			
Chiropractor				
No (n = 1,038)	1	.041	475	
Yes (n = 309)	.76 (.58, .99)			

All assumptions were met (HR <1 means longer time until end benefits, reduced rate of ending benefits)

*uHRR* univariable hazard rate ratio, *aHRR* adjusted hazard rate ratio

Form8v99 = healthcare provider form version 1999, Form8v03 = healthcare provider form version 2003

### Time on Benefits for First Compensated Back Pain Episode

The mean duration of time on disability benefits for our sample was 128 days (95 % CI 119–137) from inception point for this analysis (4 weeks after injury) [median = 57, (95 % CI 54–60)]. Almost a third (32.0 %) of workers was still on benefits at 3 months, 15.2 % at 6 months, 8.7 % at 12 months and 6.6 % at 2 years post-injury.

Table 2 shows descriptive statistics for predictive factors, as well as the univariable and multivariable association with the outcome of time on benefits in the first episode.

Predictive factors for longer time on benefits were: older age, greater physical demands in the workplace, having a prescription for opioids reimbursed by the WSIB, employer doubt about work-relatedness of the back injury, and poor recovery expectations by the HCP. Union membership, having an early RTW program in the workplace, participating in a work rehabilitation program and communication of functional abilities by the HCP were protective factors for longer time on benefits. Interaction terms—age × workplace accommodation and prior sick leave × workplace accommodation [27]—were not statistically significant and did not improve model fit. The AUC of the prediction rule for time on benefits was .71 (95 % CI .67–.75) at 6 months and .79 (95 % CI .74–.84) at 24 months.

The HRR increased from 1.51 (95 % CI 1.29, 1.78) for the second risk quartile, to 1.90 (95 % CI 1.64, 2.22) for the third risk quartile to 2.66 (95 % CI 2.27, 3.12) times increased duration of benefits compared to those in the lowest risk quartile. The median number of days on benefits increased from 41 days in the lowest risk quartile, to 52 in the second risk quartile, to 66 in the third risk quartile and to 88 days in the highest risk quartile. The survival curves for the four risk categories can be found in a Supplemental Figure.

A post hoc comparison of cases with and without missing data did not show significant differences with respect to key factors in the final models, or on outcomes. Although a slightly worse fit of the model was found based on complete cases, the fit statistic for time-on benefits outcome with 24-month follow up remained in the fair category (AUC = .75 at 24 months).

### Time Until Recurrence After First Episode

The mean number of days until a recurrence for those still on benefits at 4 weeks and at risk for a recurrence during follow-up (n = 1,347) was 547 days (95 % CI 532–562). The median number of days was not calculated because of the percentage of censored cases at the end of follow up. 1,012 workers (75.1 %) had not experienced a recurrence 2 years after the first day of injury; 89.1 % of cases had no recurrences at 30 days, 81.9 % of cases had none at 3 months, 78.3 % of cases had none at 6 months, and 76.3 % of cases had none after 12 months.

Table 3 shows descriptive statistics for predictive factors and their univariable and multivariable association with time until recurrence. Of the 25 factors considered, 17 had an association with a *P* < .20 and were entered into our bootstrapping analysis. The following five variables that increased the time until a recurrence were retained in the final multivariable model: time on benefits in the first episode, older age, male sex, manual work, opioid prescriptions reimbursed by WSIB, and no communication of ability to RTW by the HCP. The AUC of the prediction rule for time until recurrences was .60 (95 % CI .54, .64) at 1 month, .61 (95% CI .57, .65) at 3 months, and .61 (95 % CI .57, .65) at 6 months after the end of the first episode on benefits. We did not generate survival curves and hazard rate ratios between risk categories for this outcome because of the poor ability of the model to discriminate, as shown by the AUC values.

## Discussion

### Summary of Main Findings

The following factors were predictive of a longer time on benefits: older age, greater physical demands in the workplace, employer doubt regarding the work-relatedness of the back injury, and receiving a prescription for opioids reimbursed by the WSIB during the first 4 weeks of the claim. The following factors were predictive of a shorter time on disability benefits, union membership, availability of an early RTW program, positive recovery expectations on the part of health-care providers, being entered in a work rehabilitation program, and communication of functional ability to RTW by the HCP. Our final model demonstrated fair predictive accuracy [29].

The factor predictive of a longer time until recurrence was male gender. Factors predictive of a shorter time until recurrence were greater physical demands in the workplace, receiving a prescription for opioids, and communication of functional ability to RTW by the HCP. The time on benefits in the first episode was not associated with time until recurrence, but was included in the model a priori. This final model had poor predictive accuracy.

### How Does this Study Compare to Other Studies?

The model fit for time on benefits was comparable to the fit presented in other studies on BP that reported a discriminative ability of .80 [31] and .76 [32]. Predictive accuracy of the model was better compared to others reporting an AUC of .63 [10] and .69 for the Örebro Musculoskeletal Screening Questionnaire in a Canadian workers’ compensation setting [33].

*Maher* states that “our current understanding about BP perhaps makes more accurate prediction impossible” [11]. Pransky et al. [18], however, argue that, although only 12 % of overall variance was explained by their model, high-risk and low-risk tertiles were readily distinguished. Explained variance does not seem to be an appropriate way to report model fit in prediction when using survival analysis, and the AUC is a better method to describe model fit [30].

In our final model, older age was associated with greater time on disability benefits, but not for time until recurrence. Some argue that age is not a useful factor since it is ‘non-modifiable.’ However, age is still relevant when communicating prognosis to a patient and setting expectations. Moreover, one study has found, in a post hoc subgroup analysis, that older workers benefited from a RTW intervention more than younger workers [27]. Our study did not confirm an interaction between workplace intervention and older age.

### Relevance of the Findings

A decision tool based on our study may be helpful to those working in work disability prevention. Decisions are often made by case managers who are facing time constraints while trying to process an overload of information. They could use a decision tool to make an evidence-based first selection of cases at higher risk of being on benefits for an extended period, and refer these cases to interventions that improve the likelihood of RTW [34, 35]. Before being put into practice, any prediction rule should be compared to usual care to evaluate its impact on relevant outcomes [14].

### Study Strengths and Limitations

A study strength is that the model is based on data routinely collected by the WSIB. As such, limited additional resources would be needed to implement the prediction rule in practice.

A second strength is that data was collected from different stakeholders in the RTW process [1], whereas previous research often relied on data collected from a single perspective, mostly that of the injured worker or patient.

Predictive models based on one dataset can be overly optimistic in estimating the predictive value. Prior knowledge summarized in a systematic review on this topic and internal validation techniques were used to obtain models that are more likely generalizable to future populations [35]. Bootstrapping is a relatively new method of validation, comparable to split half validation. Bootstrapping mimics the process of sampling from the underlying population. Bootstrap samples (n = 2,000) are drawn with replacement from the original sample to introduce a random element [16]. The resulting effect estimates from all 2,000 models were pooled to generate more stable effect estimates. Validation in a different dataset either from a different time frame or jurisdiction is, however, preferred. The current model will likely be overfitting the data somewhat, despite the relatively large sample size. We will validate this prediction rule in a prospective cohort study in a similar population that is currently underway. Generalizing findings from similar studies from one jurisdiction to another has shown to be difficult. A validation study in The Netherlands for instance showed that a rule developed in the USA [36, 37] did not improve outcomes in the Dutch setting. Geographical and temporal validation of established predictive factors is therefore required in each specific context [16].

We included a missing category in our model because multiple imputation was not possible in this dataset, since the basic assumptions were not met. This choice only affects a limited number of variables. The factor “Union member” has 176 missing and this category is significantly associated with faster RTW. This variable was extracted from worker report. Those workers that had a fast RTW probably did not have the need to send in a worker’s form. A similar mechanism could explain the missing data (n = 122) for the information on having an early RTW program: those that were expected to RTW soon were likely not offered an RTW program because it was not needed and therefore nothing was reported.

HCPs’ recovery expectations were significantly associated with time on benefits in our multivariable model, even though many workers did not have data on this factor because HCP forms were changed in 2003. The new 2003 form did not contain this information, but many HCPs were still using the old 1999 forms in 2005. Our findings and the available evidence [17, 38] suggest that WSIB should consider re-initiating practices to capture recovery expectations.

One study limitation is that questionnaires were not scientifically validated, at least not beyond the criterion of face validity. It is not feasible for a workers’ compensation board to send out lengthy, burdensome surveys to their stakeholders where data were only used for administrative purposes.

We selected predictors available in the dataset for inclusion in our model based on a systematic review of studies that explored predictors of time on benefits/sick leave following a first episode of BP [17]. We selected the same factors to explore predictors of recurrence due to a lack of good quality studies on recurrences. It is no surprise, then, that time on benefits during the first episode was predicted with more accuracy than recurrences were. A change in design would probably improve the model fit: prognostic information should be collected closer to the inception point that is different for this outcome (at the time the first episode ends) where our study was based on data collected during the first 4 weeks after injury.

### Alternative Explanations for the Findings

The role of opioids in the management of BP has been debated [39]. A number of studies have looked at the prognostic value of opioid use in work disability [17, 39]. In our study, opioid prescriptions paid for by the WSIB are an indicator for poor outcome. A limitation to this measure is that not all opioid use was captured since some prescriptions were paid through workplace health coverage. Therefore, the factor might be a surrogate indicator for lacking health-care benefits in specific workplaces. Another possible explanation is that this measure is a surrogate indicator for severity of BP, which is not captured by other measures. This does not imply that prognosis improves when opioids are not reimbursed; however, it could be used to flag more complicated cases requiring additional intervention to improve outcomes. Data from this study were collected before the Canadian Guideline for Safe and Effective Use of Opioids [40] was implemented, which may impact results. Data reported by the HCP on the prescription of any medication did not show an association with outcomes, and might be too imprecise for prediction purposes since it lacks specific reference to opioids.

The impact of having a RTW program in the workplace on time on disability benefits is plausible when taking into account the available evidence [41]. After questions from our stakeholders, further analysis (see Supplement) showed that RTW programs are not limited to larger companies (some larger companies reported not having a RTW program in place, while some smaller workplaces (<50 FTE) reported they did). The predictive power of having a RTW program was greater than the effect of workplace size.

We interpreted the availability of FAFs as a means for the health-care provider to communicate functional ability to RTW. A HCP likely sends FAFs in the weeks leading up to an expected RTW date. A HCP would most likely not send a FAF when no RTW is expected in the first 4 weeks. Recovery expectations and communication of functional ability to RTW, however, were not correlated in cases that had data available on both factors (n = 235).

### Suggestions for Future Research

Our analyses show that predictive factors for time on benefits during a first BP-related workers’ compensation claim are not necessarily the same as those for recurrences. Both outcomes share physical demands, opioid use and communication of physical abilities as predictors. More exploratory research is needed to better capture the complexity of the topic and challenges in design when studying recurrences in this field.

The accuracy of our predictive models might be improved by adding information on established prognostic factors like injury severity, either by a more precise diagnosis code (radiating pain versus other) or information on pain rating [17] and functional disability as measured with validated measurement tools [13, 17].

## Conclusion

Time on workers’ compensation benefits following a first episode of work-related BP can be predicted with fair accuracy by using data routinely collected by a workers’ compensation board. Time until recurrence (i.e. going back on benefits due to work-related BP) after the initial episode can only be predicted poorly. Future research on prediction rules should focus on evaluating the effectiveness of using these rules in daily practice compared to usual care.

## Electronic supplementary material

Below is the link to the electronic supplementary material.

Supplementary material 1 (DOCX 18 kb)

Supplementary material 2 (DOCX 115 kb)

Supplementary material 3 (DOCX 15 kb)
