# Supplementary material for: Predicting Time on Prolonged Benefits for Injured Workers with Acute Back Pain
Source: J Occup Rehabil. 2014 Aug 28;25(2):267–78. doi: 10.1007/s10926-014-9534-5 (PMC4436678; doi:10.1007/s10926-014-9534-5)
Supplement: Supplementary file 1 — Supplementary material 1 (DOCX 18 kb) [file 10926_2014_9534_MOESM1_ESM.docx]

Online tables:

Supplement to Table 2: Variables not associated with time on benefits (n=1442)

| First block risk factors | uHRR | p-value |
| --- | --- | --- |
| Years of experience: Missing (n=437)  < 1 year (n=130)  > 1 yr experience (n=96)  > 2 years (n=779) | 1.03 [.85, 1.24]  1.03 [.82, 1.28]  1 | p=.98 |
| Previous claim, yes (n=1091)  no (n=351) | 1.07 [.94, 1.21]  1 | p=.343 |
| Earnings (n=1442) | 1 [1.00, 1.00] | P=.647 |
| Prior similar injury | 1.04[.92, 1.17] | p=.530 |
| Worker report previous claim: WCB (n=341)  Other (n=47)  Missing (n=1054) | .92 [.81, 1.05]  .88 [.65, 1.19]  1 | p=.341 |
| Restrictions operating motor vehicle, No (n=72)  Yes (n=201)  Missing (n=1169) | 1  1.20 [.91, 1.59]  1.06 [.83, 1.35] | P=.22 |
| Task limitations: No limitation (n=23)  < 2limitations (n=193)  >2 limitations (n=340)  No RTW (n=425)  Missing (n=347) | 1.55 [1.01, 2.38]  1.03 [.87, 1.23]  1.01 [.87, 1.17]  1  1.08 [.93, 1.25] | P=.358 |
| Medication Prescribed: Yes (n=353)  No (n=733)  Missing (n=356) | 1  1.05 [.92, 1.20]  1.10 [0.94, 1.28] | P=.478 |
| Have you discussed RTW: No (n=77)  Yes (n=735)  Missing (n=630) | .85 [.66, 1.08]  1  .94 [.85, 1.05] | P=.299 |

All assumptions were met. (HR<1 means longer time until end benefits, reduced rate of ending benefits. uHRR=univariable Hazard rate ratio, aHRR=adjusted hazard rate ratio). Form8v99= the healthcare provider form version 1999. Form8v03 = the health-care provider form version 2003. *Form8v99= the healthcare provider form version 1999. Form8v03 = the healthcare provider form version 2003.

Supplement to Table 3: Variables not associated with time until recurrence (n=1347)

| First block risk factors | uHRR | p-value |
| --- | --- | --- |
| Years of experience: < 1 year (n=127)  > 1 yr experience (n=87)  > 2 years (n=731)  Missing (n=402) | .94 [.64, 1.40]  1.30 [.86, 1.96]  1  1.11 [.87, 1.41] | .548 |
| Previous lost-time claim: Yes (n=736)  No (n=611) | 1.12 [.90, 1.39]  1 | .297 |
| Previous no-lost-time claim: Yes (n=704)  No (n=643) (ref) | 1.14 [.92, 1.41]  1 | .238 |
| Gross earnings | 1.00 [1.00, 1.00] | .25 |
| Language: French/English (n=1306)  Other (n=41) | 1  1.06 [.58, 1.93] | .856 |
| Union member: Yes (n=583)  No (n=596)  Missing (n=45) | 1.11 [.88, 1.40]  1  1.26 [.71, 2.22] | .573 |
| Prior similar injury: Yes (n=389)  No (n=694)  Missing (n=141) | 1.13 [.88, 1.44]  1  1.10 [.77, 1.58] | .620 |
| Worker report previous claim  WCB (n=316)  Other (n=43)  Missing (n=865) | 1.05 [.81, 1.36]  1.46 [.85, 2.51]  1 | .419 |
| Employer continued salary: Yes (n=178)  No (n=114)  Missing (n=1031) | 1.05 [0.63, 1.74]  1  1.23 [0.81, 1.87] | .415 |
| Doubt work relatedness: No (n=978)  Yes (n=182)  Missing value (n=173)  Missing Form 7 (n=14) | 1  1.29 [0.96, 1.74]  1.12 [0.82, 1.55]  - | .241 |
| Worker signed: Yes (n=303)  No (n=1016) ref  Missing (n=14) | 1.09 [0.85, 1.41]  1  1.23 [0.46, 3.30] | .755 |
| Recovery expected: Yes (n=215)  No (n=7)  Missing (n=37)  Form 8v03 Present (n=1008)  No Form 8 (n=80) | 1  .62 [.09, 4.51]  1.34 [.70, 2.59]  1.14 [.83, 1.55]  - | .697 |
| Restrictions operating motorvehicle: No (n=211)  Yes (n=18)  Missing (n=30)  Form 8v03 Present (n=1008)  No Form 8 (n=80) | 1  .91 [.33, 2.52]  1.02 [.46, 2.25]  1.09 [.80, 1.48]  - | .933 |
| Use public transport, No (n=11)  Yes (n=207)  Missing (n=41)  Form 8v03 Present (n=1008)  No Form 8 (n=80) | 1  .81 [.25, 2.60]  .75 [.20, 2.78]  .88 [.28, 2.76]  - | .904 |
| Task Limitations: 0 (n=22)  1 (n=181)  2 (n=314)  3 (n=394)  Missing (n=110) | 1  .91 [.36, 2.30]  1.24 [.50, 3.05]  1.04 [.42, 2.56]  .98 [.38, 2.56] | .515 |
| Medication Prescribed: No (n=681)  Yes (n=331)  Missing (n=9)  Form 8v99 Present (n=248) | 1  1.16 [0.90, 1.51]  1.44 [0.46, 4.52]  0.98 [0.72, 1.32] | .597 |
| Have you discussed RTW: No (n=71)  Yes (n=693)  Missing (n=257)  Form 8v99 Present (n=248) | 1  0.79 [0.51, 1.23]  0.69 [0.42, 1.13]  0.72 [0.44, 1.18] | .487 |
| POC: No (n=1094)  Yes (n=253) | 1  1.01 [0.76, 1.33] | .962 |

- All assumptions were met. (HR<1 means longer time until end benefits, reduced rate of ending benefits. uHRR=univariable Hazard rate ratio, aHRR=adjusted hazard rate ratio). Form8v99= healthcare provider form version 1999. Form8v03 = healthcare provider form version 2003.
