# Supplementary material for: Predicting Time on Prolonged Benefits for Injured Workers with Acute Back Pain
Source: J Occup Rehabil. 2014 Aug 28;25(2):267–78. doi: 10.1007/s10926-014-9534-5 (PMC4436678; doi:10.1007/s10926-014-9534-5)
Supplement: Supplementary file 2 — Supplementary material 2 (DOCX 115 kb) [file 10926_2014_9534_MOESM2_ESM.docx]

Supplemental figure: Survival curves of risk categories for end of benefits prediction model
