# Supplementary material for: Predicting Time on Prolonged Benefits for Injured Workers with Acute Back Pain
Source: J Occup Rehabil. 2014 Aug 28;25(2):267–78. doi: 10.1007/s10926-014-9534-5 (PMC4436678; doi:10.1007/s10926-014-9534-5)
Supplement: Supplementary file 3 — Supplementary material 3 (DOCX 15 kb) [file 10926_2014_9534_MOESM3_ESM.docx]

Supplemental data:

**Examine firm size and compare with the presence of a RTW program**

Firm size and RTW programs are correlated (p=0.29) but not identical. We extracted firm size based on FTEs in 2004 and 2005 and examined them in three categories: < 50 in both years, >50 in both years, >50 in one year but not the other. In the table below, you can see that over 50% of the smaller companies report having a RTW program, and almost 10% of the bigger companies report that they do not have a RTW program.

|  | **Firm size 0= < 50 in both years, 1 = <50 in either year, 2 is > 50 in both years** | | | Total |
| --- | --- | --- | --- | --- |
|  | < 50 in both years | < in 1 year => 50 in other year | >= 50 in both years |  |
| No RTW program | 192 | 9 | 77 | 278 |
| Yes, RTW program | 362 | 19 | 655 | 1036 |
| Missing | 53 | 4 | 63 | 120 |
| Total | 607 | 32 | 795 | 1434 |

In our univariate analysis, not corrected for any other factor, we found a statistically significant association between firm size of 50 FTE with time until end of benefits .

| **Variables in the Equation** | | | | | | | | |
| --- | --- | --- | --- | --- | --- | --- | --- | --- |
|  | B | SE | Wald | df | Sig. | Exp(B) | 95.0% CI for Exp(B) | |
|  |  |  |  |  |  |  | Lower | Upper |
| FTEbothyears |  |  | 6.358 | 2 | .042 |  |  |  |
| FTEbothyears(1) | .128 | .190 | .450 | 1 | .502 | 1.136 | .782 | 1.651 |
| FTEbothyears(2) | .140 | .056 | 6.293 | 1 | .012 | 1.150 | 1.031 | 1.283 |

No association was found when the cut-off of 20 FTE was used (workplaces larger than 20 FTE need to have a joint health and safety committee in place in Ontario).

When adjusting for possible other important factors, the effect size of firm size lost statistical significance. Having an early RTW program does remain statistically significant and has a substantial effect size. This is in line with what we know from the literature. After examining this hypothesis, we therefore decided to use the question on having a RTW program in the workplace and NOT firm size, because they are sufficiently different.
